# Hollow and porous TiO_2_ in PVA matrix nanocomposite green synthesis using ionic liquid micelle for Congo red removal from contaminated water

**DOI:** 10.1038/s41598-022-24068-x

**Published:** 2022-12-06

**Authors:** Arsalan Shahabadi, Behrang Golmohammadi, Hemayat Shekaari

**Affiliations:** Department of Physical Chemistry, University of Tabriz, Tabriz, Iran

**Keywords:** Green chemistry, Materials chemistry, Physical chemistry, Theoretical chemistry

## Abstract

A new green procedure has been applied to prepare TiO_2_ nanocomposite in polyvinyl alcohol (PVA) matrix using an aqueous micelle solution of ionic liquid 1-methyl-3-octylimidazolium bromide by determining critical micelle concentration (CMC). The COSMO-SAC model has been used to calculate the activity coefficient of water and understand the water molecules’ behavior in the synthesis mixture. The prepared nanocomposite was porous and layered that has been characterized using FT-IR, XRD, DSC, TGA, SEM, EDX, and elemental mapping. The prepared nanocomposite has been used to remove Congo red dye from contaminated water with the adsorption process. The Langmuir, Freundlich, and Temkin isotherms have been used for modeling equilibrium adsorption of dye removal. Also, the optimized process factors have been evaluated that could achieve 97% dye removal in the following conditions: *pH* = 12, *T* = 25 ℃, and *t* = 45 min using 0.2 g TiO_2_@PVA (Mesh 100)/L of 10 ppm Congo red aqueous solution. Also, the efficiency of the nanocomposite was 88% after 5 recovery cycles from the optimized condition.

## Introduction

Nanocomposites are an important part of the developed technologies in the present time. Synthesis of nanocomposites requires a lot of practice, and consuming hazardous materials in the synthesis procedure is inevitable. Green synthesis of nanocomposites is a recent challenge considering the endangered world^1^. Ionic liquids are green materials that are replacing old hazardous materials. Hydrophobic ionic liquids could be used to prepare aqueous micelles solutions^2–4^. The micelle solutions have the potential to form rheological stable nanofluids^5^.

The micelles form a different phase in the solvent bulk and usually, their density is lower than the solvent that led to the floating of the micelles. Dispersed nanoparticles in the shell of the micelle solution could be stabilized the corresponding nanofluid more than regular solvent. It is possible to prepare the polymeric nanocomposite using micelle nanofluid.In this respect, imidazole-based ionic liquids are considered one of the most efficient series of ILs families. The length of the alkyl chain in the ionic liquids 1-alkyl-3-methylimidazolium halide is the most efficient factor in the formation of the micelle, and the higher length of the alkyl chain makes micelle formation occur in a lower concentration^6^.

Preparing aqueous TiO_2_ nanofluid has been established using ethylene glycol or other organic solvents^7^. However, organic solvents have various problems such as volatility and toxicity. Ionic liquids could overcome these problems and have other advantages such as higher thermal conductivity^8,9^. Also, polyvinyl alcohol is a water-soluble polymer that could be used to prepare fully aqueous-based nanocomposite with TiO_2_ ionic liquid aqueous nanofluid^10^.

Using nanocomposites is one of the common methods to process wastewater. Dye removal using nanocomposite is one of the most efficient methods that are used to treat wastewater^11^. Congo red is one of the industrial dyes that are used on an industrial scale. However, removing the Congo red is one of the challenging processes in wastewater treatment due to its pH dependency and high-water solubility. However, it has been shown that using nanocomposite could be efficient in removing Congo red from wastewater^12^.

In the present work, the conductometric method has been utilized to determine the critical micelle concentration of ionic liquid 1-methyl-3-octylimidazolium bromide^13,14^. Afterwards, the nanofluid of TiO_2_ has been prepared in the micelle solution of the IL and is used to synthesize the PVA nanocomposite with the phase inversion technique. The stability of the nanofluid has been investigated using viscosity measurement during the time after the dispersion of TiO_2_ nanoparticles. The prepared nanocomposite has been analyzed using FT-IR, TGA, DSC, and SEM. Also, it has been used to investigate the Congo red adsorption from aqueous solution, and different adsorption models such as Langmuir, Freundlich, and Temkin have been used to comprehend the adsorption mechanism.

## Results and discussion

### Determination of critical micelle concentration (CMC)

**Figure 1:**
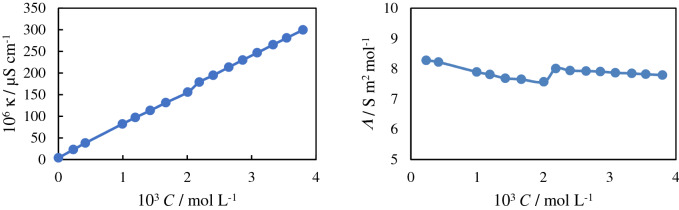
The (**a**) conductance and (**b**) molar conductivity of aqueous solutions of [MOIM][Br] under atmospheric pressure at 25 °C.

The conductance and molar conductivity of different concentrations of aqueous solutions of the IL under atmospheric pressure at 25 °C are given in Fig. 1. The results show that the *CMC* is about 0.002 mol L^−1^. Determination of the *CMC* is the most important part of this investigation.

**Figure 2:**
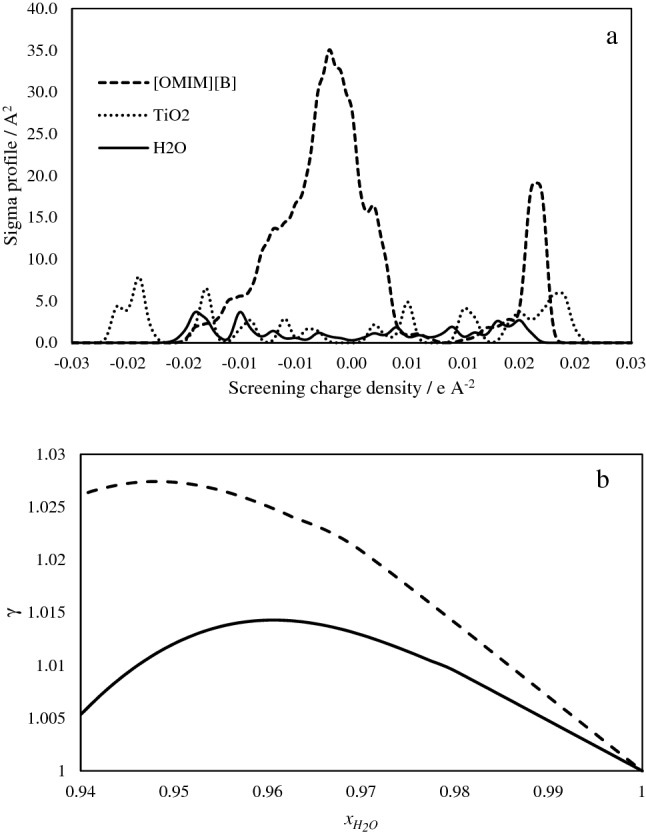
The results of COSMO-SAC modeling for the nanofluid containing water + IL + TiO_2_: (**a**) sigma-profiles of the components, (**b**) water activity coefficient plot against its mole fraction in 0.01 (solid line) and 0.02 (dashed line) mol fraction of TiO_2_ at 25 °C.

The activity coefficient is a measure of the tendency of a species to escape from a phase. Accordingly, the escaping of a water molecule in an aqueous solution of the IL in presence of TiO_2_ could be used to interpret the *CMC* behavior. The COSMO-SAC is a quantum mechanics-based model that is used to evaluate the activity coefficient of water in the mixture^15^. The σ-profiles of the species have been evaluated using the dmol3 module of (Biovia materials studio, 2020) with the functional of GGA-VWN-PB^16^. The evaluated σ -profiles are given in Fig. 2-a that are used to calculate the activity coefficient of water in the aqueous solution of the IL in the presence of 0.01 and 0.02 mol fractions TiO_2_ at 25 °C, and the results are given in Fig. 2-b. The IL has an intensive sigma profile due to its charged species, and the cation has a wide range of charge density on the left side of the σ-profile that affects the polarity of the component. On the other hand, the activity coefficient of water increases with the addition of the IL up to the CMC and then decreases. Also, with the addition of the TiO_2_ to a mole fraction of 0.02 the activity coefficient of water increases. The water molecules tend to go away from the interacted micelle and the TiO_2_ particles while an increment in the concentration of the IL increases the local micelle density and sinking of the micelle to the bottom beside the TiO_2_ particles and dispersion fails. According to the results, twice concentrated micelle solution (twice of CMC) of the IL is used to prepare the nanofluid of the TiO_2_ with a concentration of 0.005 mol kg^−1^ as the stock fluid for preparation of the nanocomposite. The prepared nanofluid was dispersed using ultrasonic propagation. The upper limit for the IL concentration before the reduction of water activity coefficient is the mole fraction of 0.03 based on the COSMO-SAC results.

### Stability of the nanofluid

**Figure 3:**
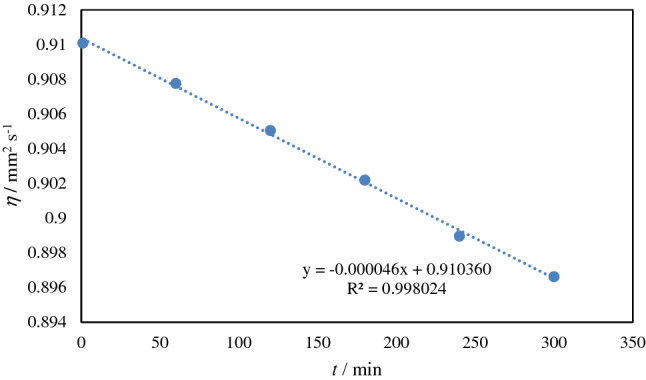
Variation of kinetics viscosity of the nanofluid 0.005 mol kg^−1^ TiO_2_ in aqueous 0.004 mol kg^1^ [MOIM][Br] at different time intervals after the dispersion at 25 °C.

The results of the stability of the nanofluid have been determined from the slope of the kinetics viscosity measurement over the time up to 5 h. The sample was containing 0.005 mol kg^−1^ TiO_2_ dispersed in 0.004 mol kg^−1^aqueous solution of the IL. The results of the kinetic viscosity are given in Fig. 3. As could be seen the slope is − 0.000046 mm^2^ s^−2^ which means the prepared nanofluid was quite stable for 5 h after dispersion which gives us enough time for processing of the nanocomposite with aqueous nanofluid.

### Characterization of the TiO2@PVA nanocomposite

#### FT-IR spectrum

**Figure 4:**
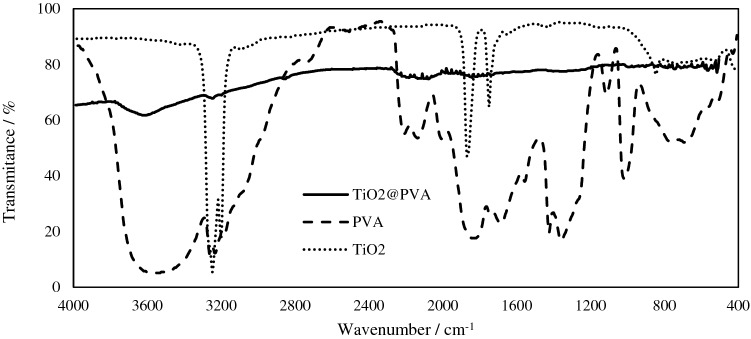
The FT-IR spectrum of nanocomposite TiO2@PVA (solid line), PVA (dashed line), and TiO2 (pointed line).

The FT-IR spectra of the PVA, TiO2, and nanocomposite have been given in Fig. 4. As could be seen the intensive vibrational peaks of the two components have been weakened by forming the nanocomposite. The observed limitation on the vibration’s intensity is due to the newly formed structure of the nanocomposite and the Walden forces between the polymer and nanoparticle interactions. Also, the annealing of the nanocomposite probably led to cross-linking between the polymer chains and traps the nanoparticles that both limits the vibrations in the structure.

#### XRD pattern

**Figure 5:**
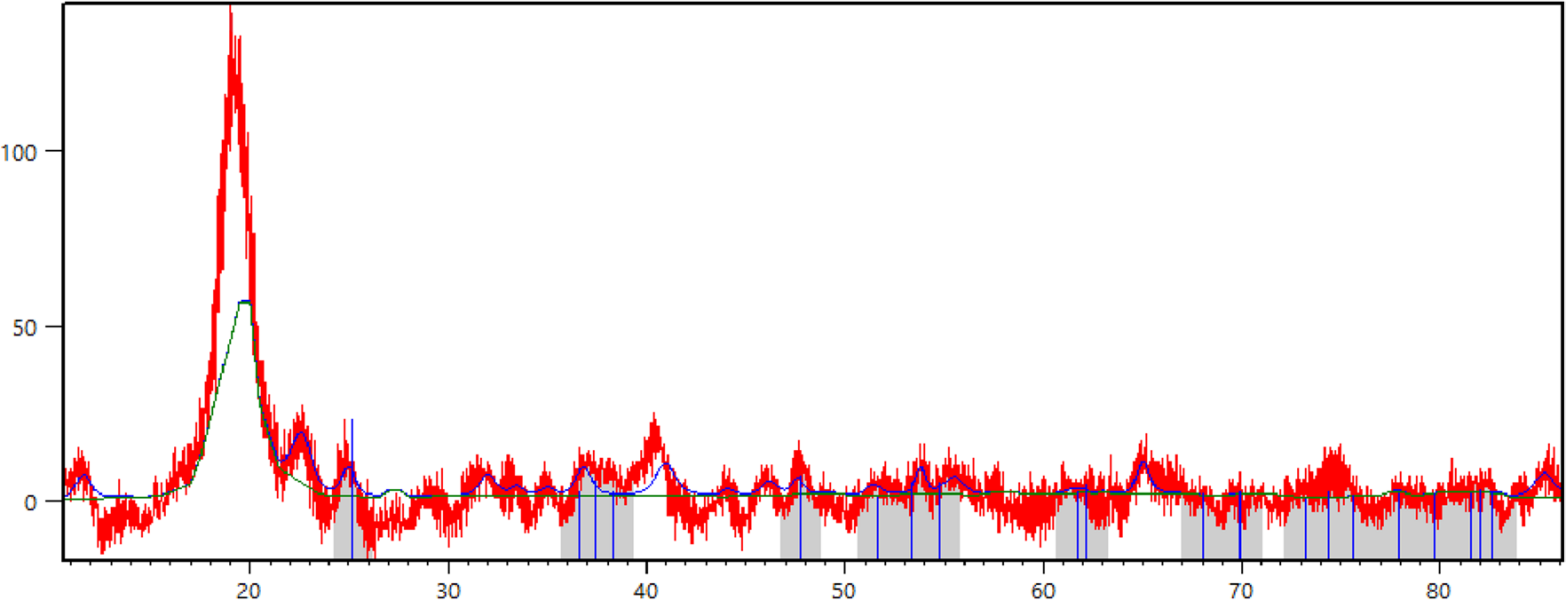
The XRD pattern of the synthesized nanocomposite TiO_2_@PVA with red line pattern and blue peaks for anatase TiO_2_.

The XRD pattern of the synthesized nanocomposite has been given in Fig. 5. The peaks of a monoclinic unit cell of the PVA are observed as mentioned earlier in the literature^17^. Also, anatase TiO_2_ has been matched with the reference that has been highlighted in the figure. However, some additional peaks might be related to the formation of new crystalline sub-structures during the annealing. Also, the asymmetrical semicrystalline structure of residue IL in the structure of the nanocomposite might be the reason for the observed additional peaks. Since acetone could dissolve IL during the phase inversion, we don’t expect to see IL semicrystalline effect on the XRD pattern. Accordingly, thermal analysis is required to ensure the formation of new sub-structures in the nanocomposite structure.

#### Differential scanning calorimetry (DSC)

**Figure 6:**
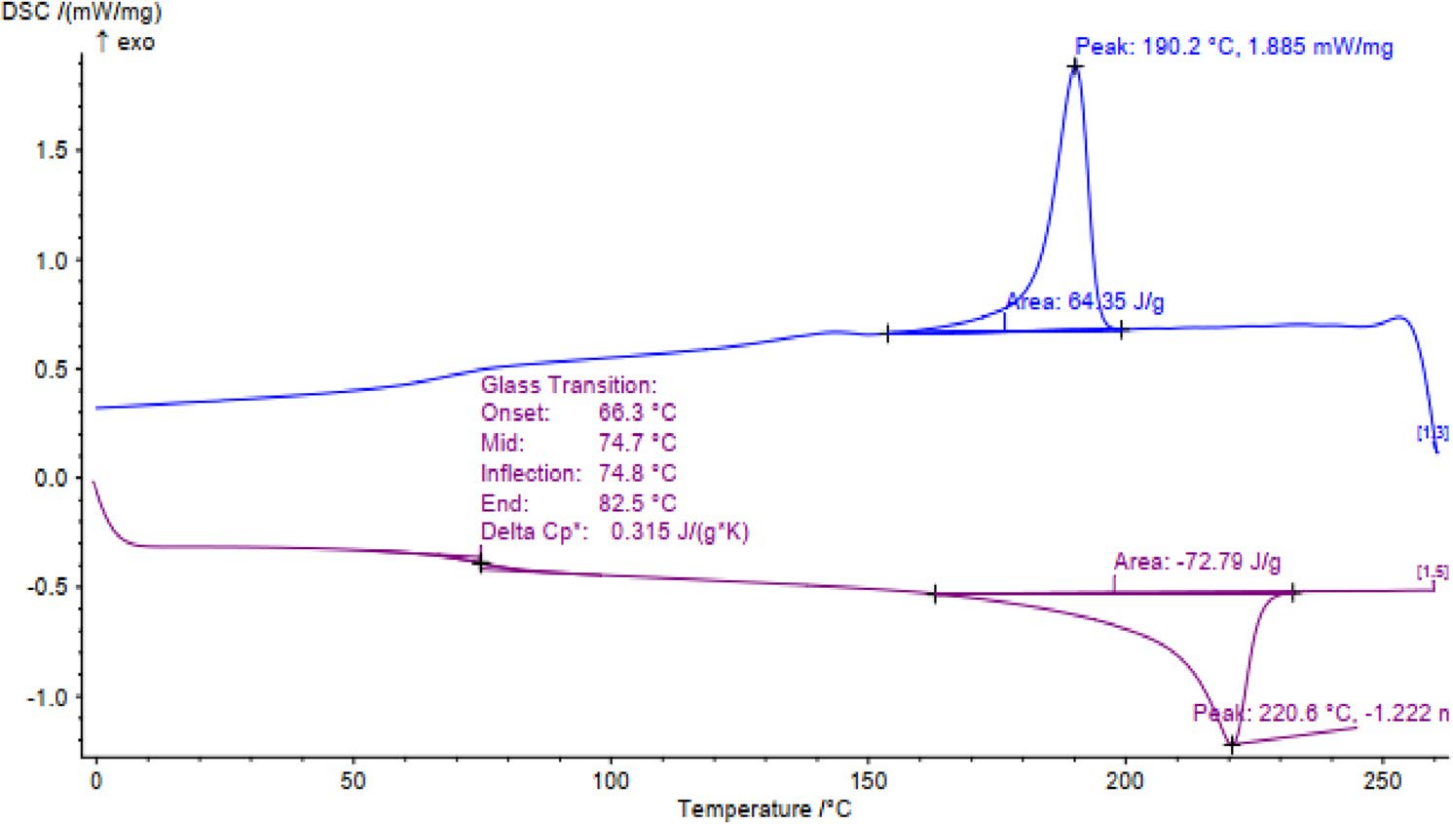
The reversible DSC analysis of the TiO_2_@PVA nanocomposite.

The reversible calorimetry results for the prepared nanocomposite are given in Fig. 6. A glass transition point has been detected for the nanocomposite at 74.75 ℃ while there was no reported transition glass temperature for pure PVA in the literature. Also, the melting point for the nanocomposite is recorded at 220.6 ℃ while the melting point of the pure PVA is at 202.7 ℃^18^. The results show a stronger structure that needs more energy to melt the nanocomposite rather than the pure PVA while the reverse cooling scanning shows a fusion point of 190.2 ℃ which means quasi-reversible and demolishing of the structure after heating. It is expected behavior in the polymeric materials due to their semi-crystalline structure with lower heat capacity rather than ionic and metallic crystal solids. Indeed, the degradation temperature of the nanocomposite structure is the major concern as a consequence of these results.

#### Thermogravimetry analysis (TGA)

**Figure 7:**
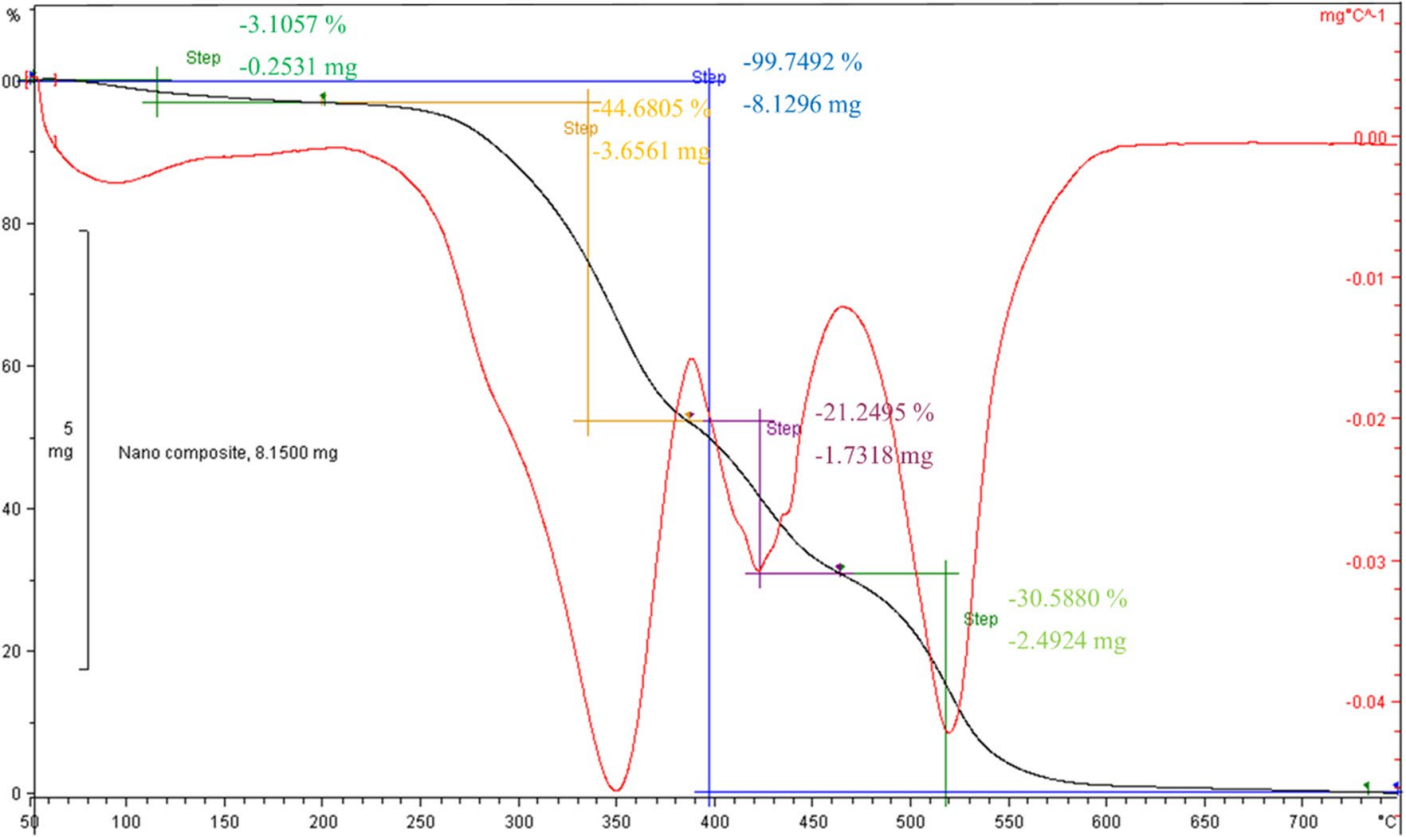
The quasi-static TGA ( −) and DTGA ( −) of the TiO_2_@PVA nanocomposite with corresponding step analysis and degradation results.

A quasistatic thermogravimetry analysis of the synthesized nanocomposite has been given in Fig. 7. This analysis has been carried out to find the degradation of the nanocomposite with temperature (thermal stability). The nanocomposite is stable up to 300 ℃ which has been recorded in two steps. Also, raising the temperature to 600 ℃ led to complete degradation of the nanocomposite. This rate was reported at about 470 ℃ for pure PVA which is below rather than the TiO_2_@PVA^19^. The results suggest that the nanocomposite would be useful and recoverable at thermal processing below 300 ℃. However, the structural features require more characterization to find out an appropriate application for the synthesized nanocomposite.

#### Scanning electron microscopy (SEM)

**Figure 8:**
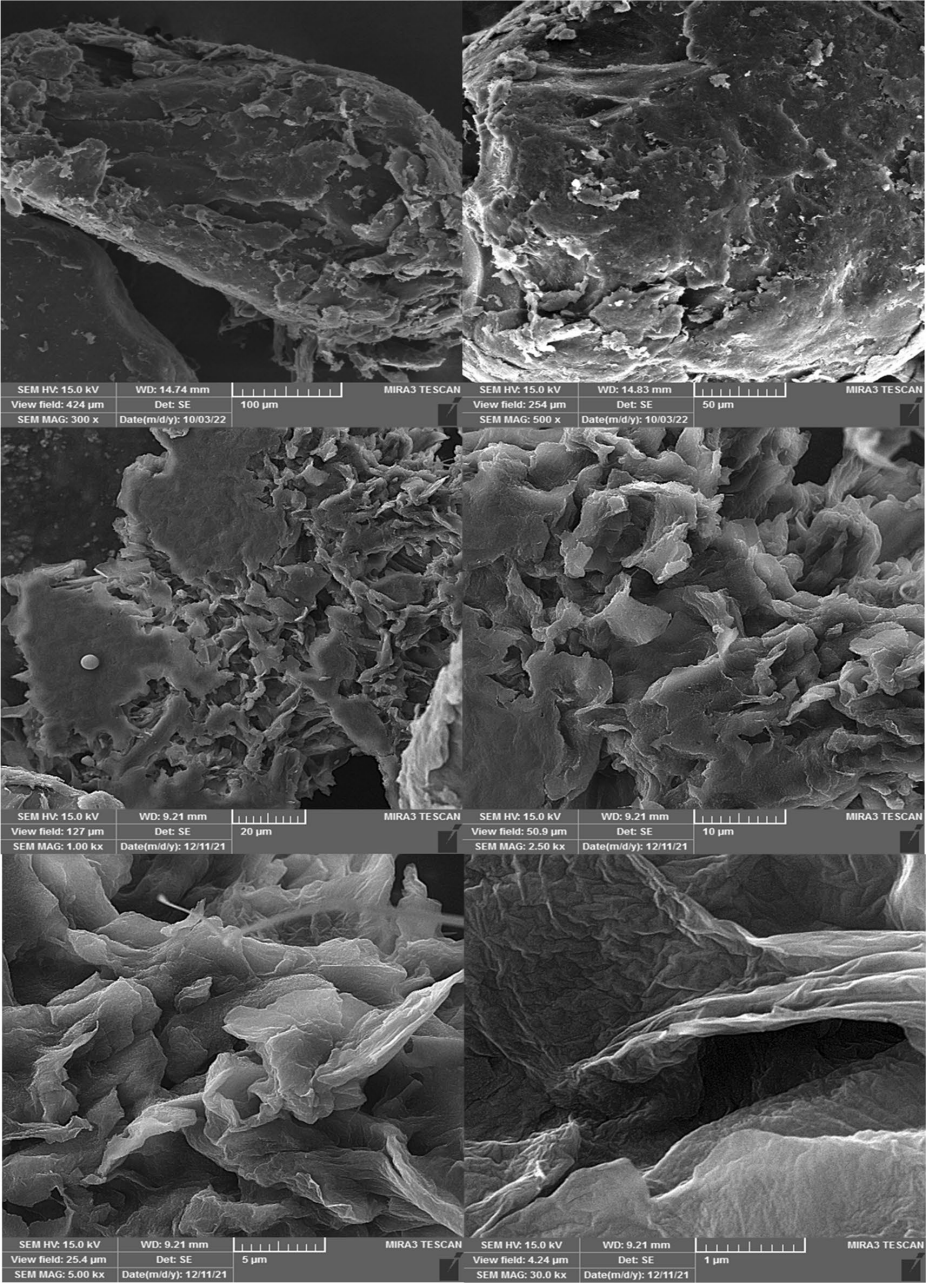
The SEM images of the synthesized TiO_2_@PVA nanocomposite with different magnifications.

**Figure 9:**
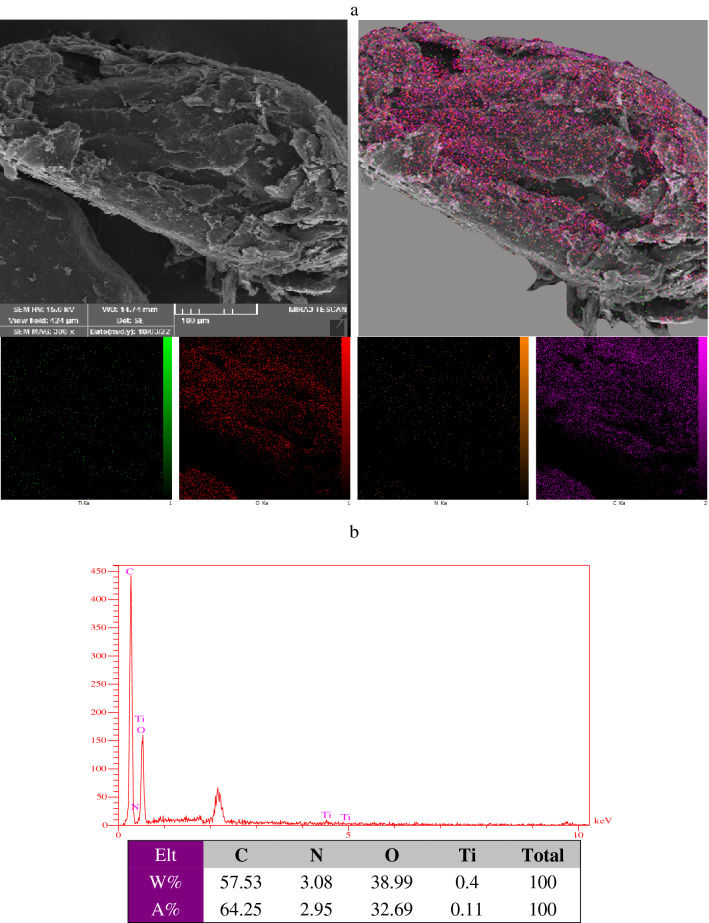
(**a**) The elemental mapping: purple spots (C), orange spots (N), red spots (O), green spots (Ti), (**b**) the EDX spectra and corresponding elemental analysis of = the TiO_2_@PVA nanocomposite.

**Figure 10:**
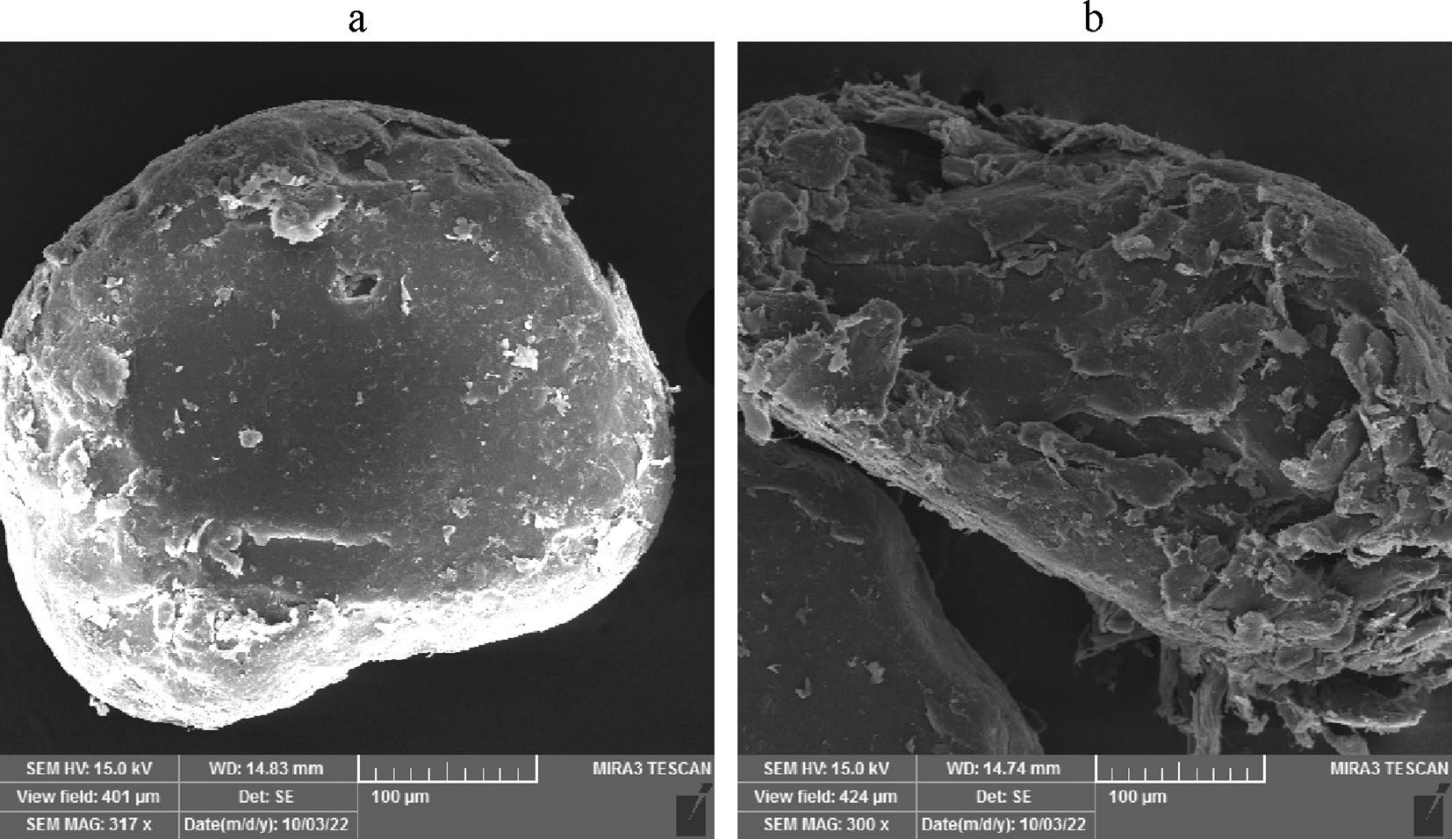
The SEM images of the nanocomposites: (**a**) without the IL in formation, (**b**) with the IL in formation.

The scanning electron microscopy images of the TiO2@ PVA are given in Fig. 8. As could be seen a layered porous structure of nanocomposite has been formed as a consequence of the phase inversion process with a hallow structure. Also, the spherical TiO_2_ has been spotted on a layer of the PVA. Probably, the structure has been formed as a consequence of existing of the IL micelles in the phase inversion process and long annealing time. However, the results suggest that the nanocomposite could provide a significant specific interface due to the existence of the nanoparticle besides a large porous volume in the polymer matrix that could be used for deposition. In Fig. 9, the elemental mapping and corresponding EDX spectra of the nanocomposite have been illustrated that confirms the formation of the nanocomposite. The nitrogen element dispersion on the structure is a proof of attachment of the ionic liquid to the structure. Also, the EDX elemental analysis and mapping show the dispersion of Ti elements on the structure that is a sign of attachment of TiO_2_ to the structure. However, the procedure to form of nanocomposite have been repeated without the IL and the corresponding SEM image has been given in Fig. 10. As could be seen the resultant nanocomposite is a bulky material while the nanocomposite using the IL in the formation is a hallow and porous material.

#### Nanocomposite synthesis mechanism

**Figure 11:**
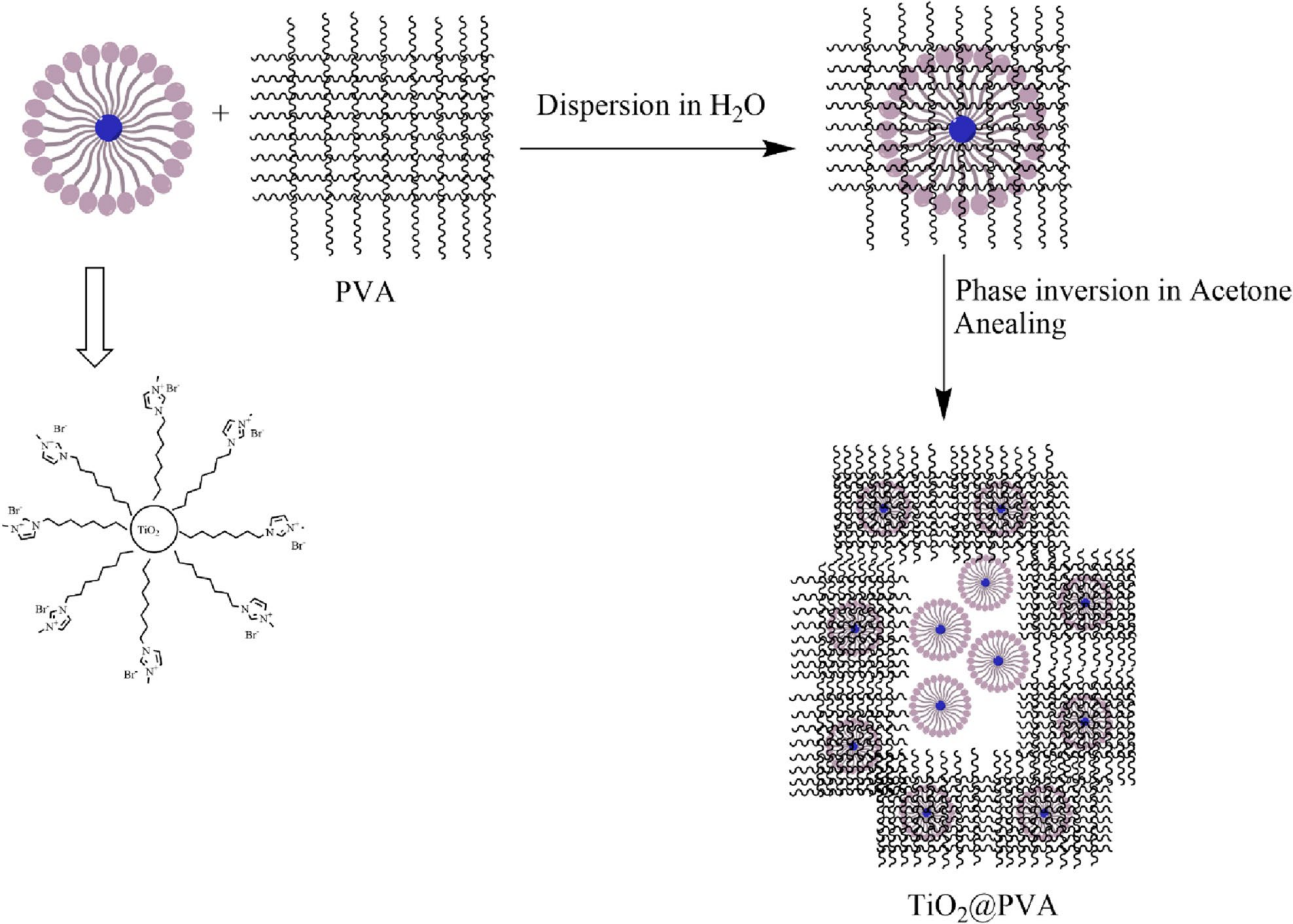
Suggested mechanism for the synthesis of hallow and porous TiO2@PVA nanocomposite.

Based on the results, it seems that the formation of hallow, layered, and porous nanocomposite is due to formation of micelle that could suspend TiO_2_ while dispersion of micelle-TiO_2_ could be placed in the bulk structure of PVA. In the stage of phase inversion, using acetone replaces the water molecules and the structure might have intention to maintain the structure with micelle-TiO_2_ attached to the bulk while it is not soluble in acetone. Further progressing would remove the water molecules completely. The suggested mechanism has been illustrated in Fig. 11. However, the phase inversion process should not take place more than 1 h due to the solubility of the IL in the acetone. This phenomenon would destroy the nanocomposite with leaking of the IL and destruction of the caged TiO_2_ in the PVA structure. On the other hand, it has been shown that without the IL the nanocomposite would be a bulky structure while in the presence of the IL it is a hallow structure. This also might be due to the micelle formation that has been removed during the phase inversion that led to from a shell while the micelles are floating in the acetone.

A combination of the characterizations suggests that the prepared nanocomposite would be a good candidate for isothermal adsorption. Accordingly, we have decided to use this nanocomposite for the dye removal process due to its porous structure and potentially thermal stable substructures with adsorption to assess the structure capability in adsorption and investigate its behavior in the adsorption according to the isotherm theories.

#### Isotherms of adsorption

The Langmuir, Freundlich, and Temkin isotherm models could be shown with the following equations, respectively:

1 $$\frac{1}{{q_{e} }} = \frac{1}{{q_{m} k_{L} }}\frac{1}{{C_{e} }} + \frac{1}{{q_{m} }}$$

2 $$\ln (qe) = \ln (k_{F} ) + \frac{1}{n}\ln (C_{e} )$$

3 $$qe = B\ln (k_{T} ) + B\ln (C_{e} )$$

**Figure 12:**
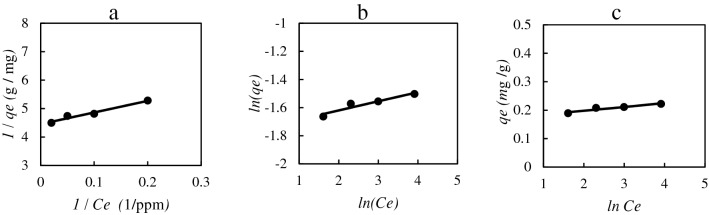
The isotherms for adsorption of the Congo red using TiO2@PVA nanocomposite: (**a**) Langmuir, (**b**) Freundlich, (**c**) Temkin.

In the presented equations, *q*_*e*_ and *C*_*e*_ are mg of adsorbed dye on the unit mass of nanocomposite (mg/g) and concentration of dye under equilibria conditions, respectively. The symbols *k*_*L*_, *k*_*F*_, and *k*_*T*_ are the constants of Langmuir, Freundlich, and Temkin isotherms, respectively. The *q*_*m*_ is the monolayer adsorbed dye concentration in the Langmuir model. The symbol *n* presented in the Freundlich model is a criterion of reversibility of the adsorption process. In the Temkin model, the B symbol is a constant related to the heat of sorption^20,21^. The results have been given in Fig. 12.

**Figure 13:**
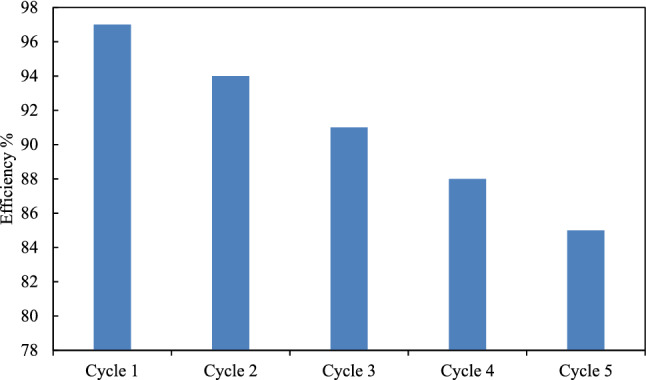
The efficiency of the TiO2@PVA nanocomposite in the optimized Congo red dye removal process after recovering in different cycles of recovery.

The results of the modelings are collected in Table 1 with the corresponding errors in parameters and average relative deviation (*ARD*) of the models. According to the *ARD* results in Table 1, the Langmuir model is the best model for the adsorption of Congo red on the *TiO2@PVA* nanocomposite with monolayer adsorption on the homogenously distributed interface. Also, the optimized condition has been evaluated considering different parameters such as pH, temperature, time, the concentration of dye, and the amount of nanocomposite adsorbent. The adsorption efficiency of Congo red raised at higher pH up to 97% dye from aqueous solution at *pH* = 12, *T* = 25 ℃, and *t* = 45 min using 0.02 g TiO_2_@PVA in 10 ml of 10 ppm Congo red aqueous solution. A lower pH reduces the efficiency of the dye removal as raising of temperature and time. However, the amount of adsorbent will increase the efficiency of dye removal from a more concentrated dye solution. The recovering ability of the nanocomposite in this process has been demonstrated in Fig. 13. The efficiency of the nanocomposite was reduced from 97 to 85% after the fifth cycle of the optimized dye removing process recovery. Indeed, the Langmuir isotherm is the best model for this process regarding its hypothesis. The reduced efficiency might be caused by different reasons such as the occupation of the pores with different solvent molecules or the dye molecules after the recovery process. The optimized removal condition has been repeated for a new nanocomposite without using ionic liquid in the formation. The removal amount at the same condition was 39% removal of Congo red that is a weak performance in the removal in the comparison of the nanocomposite with the IL in the formation.

**Table 1:** The evaluated parameters of adsorption isotherm models with the corresponding ARD from the experimental data.

Isotherm	Parameters						*% ARD*
Langmuir	*q*_*m*_ = 0.224	±	0.006	*k*_*L*_ = 1.091	±	0.003	3.695
Freundlich	*n* = 15.424	±	0.014	*k*_*F*_ = 0.174	±	0.004	5.572
Temkin	*B* = 0.013	±	0.003	*k*_*T*_ = 378,352.6	±	0.008	5.329

The obtained results for the removal of the Congo red have been compared with the literature results in Table 2. The results show that the prepared nanocomposite has significant advantages rather than the reported nanocomposites that are used to remove Congo red with adsorption method. It might be due to the hollow, layered and porous structure of the nanocomposite that could catch and store the dye molecules more efficiently.

**Table 2:** Comparison of the Congo red removal with different adsorbents with the TiO2@PVA nanocomposite.

Adsorbent	Capacity of removal/mg/g	Time for adsorption/min	Temperature/℃	References
coconut husk, raw clay, Fe(II) and Fe(II) compounds	1649.30	40	50	^22^
Mn-doped UiO-66/Aminated-graphene oxide	1265.82	40	25	^23^
MXene/PEI modified sodium alginate aerogel	3568.00	30	35	^24^
TiO_2_@PVA	3483.24	45	25	This work

### Experimental

#### Synthesis of 1-methyl-3-octylimidazolium bromide

**Figure 14:**
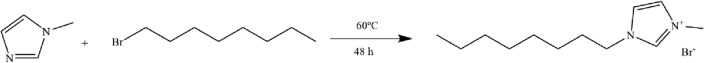
The reaction for preparation of 1-methyl-3-octylimidzaolium bromide.

An addition reaction has been used to synthesize the ionic liquid (1-methyl-3-octylimidazolium bromide; [MOIm][Br]) from 1-methylimidazole as an initial reagent. Briefly, 1.1 mmol of bromooctane was added to 1 mmol 1-methylimidazole and stirred at 273.15 K. Then reaction temperature was raised to 60 °C and stirred for 48 h^25^. The corresponding reaction is given in Fig. 14.

The crude product has been washed with ethyl acetate at least three times and the trace amount of the solvent was removed using distillation under reduced pressure. The synthesized IL was characterized using ^1^HNMR (Brucker Av-400) and the result was identical to our previous results^25^. Also, the water content of the prepared ILs was determined with volumetric Karl-Fisher titration (Metrohm, 751 GPD Titrino, volumetric method), and lower than 120 ppm water content has been detected.

#### Preparation nanocomposite (TiO_2_@PVA)

The 10 nm spherical anatase nanoparticles of TiO2 were dispersed in 0.005 M of [MOIm][Br] aqueous solution (mix1) using an ultrasonic probe (Dr. Heilsher, GmbH, UP-400,) with 100 MHz and 0.5 s sequences and 75% amplitude. A 10% *w/w* of polyvinyl alcohol (PVA) in water has been prepared (sol1). The sol1 was added dropwise to the mix1 while stirring (mix2). The mix2 was dispersed while stirring to achieve a homogenous mixture. The obtained mixture was dropwise added to the pure acetone to form a sponge-wise gel. The obtained gel has been washed with (30:70) v/ v acetone/ water several times to remove the IL. The obtained polymeric matrix was dried in an oven below 60 °C for 10 h. The annealing of the gel has been carried out in a furnace at 130 °C for 8 h. Consequently, the nanocomposite has been grinded mechanically and just 60 mesh of the particles is used for analysis and another process. It should be noted that the ball mill did not work for grinding. There is numerous reported successful nanocomposite synthesis in the literature using ultrasonic irradiation spherical shaped holmium vanadate that is used for the photocatalytic degradation of the water pollutants^26^.

#### Conductometric study and determination of IL’s CMC

The conductance of different concentrations of [MOIm][Br] in water has been measured to determine the critical micelle concentration (*CMC*) at 25 °C. The utilized instrument was Metrohm (712 Conductometer) equipped with a magnetic stirrer and circulating water thermostat (Julabo ED, Germany) with a resolution of 0.01 °C. The instrument was calibrated with a 0.01 M KCl solution that was prepared with deionized water as standard with a specific conductance of 1413 μS cm^−1^.

#### Viscometry and stability of TiO_2_ aqueous nanofluid

The nanofluid containing 0.005 mol kg^−1^ TiO_2_ in 0.004 mol kg^−1^ aqueous solution of the [MOIm][Br] has been prepared and dispersed for 30 min with an ultrasound probe with a frequency 100 kHz, with 75% amplitude and 0.5 s sequences. The stability of the nanofluid has been checked with viscosity measurements up to 5 h. An Ubbelohde-type viscometer with a proper capillary size has been used to measure the viscosity, and a water bath thermostat (Julabo ED, Germany) has been utilized for temperature stabilizing during the viscosity measurements. The sampling has been done from the upper region of the nanofluid.

#### FT-IR analysis

The FT-IR Transmittance spectra through KBr of the TiO2, PVA, and the TiO2@PVA nanocomposite have been recorded with Bruker (Tensor 270- KBr).

#### XRD analysis

The 100 mesh of the TiO2@PVA nanocomposite has been analyzed using the XRD instrument (Tongda, TD 3700, Cu Kα, λ = 1.54 Å).

#### Differential scanning calorimetry

The calorimetric measurement of the powder sample of the nanocomposite has been carried out in a reversible condition to determine the thermophysical properties such as the glass transition temperature of the synthesized nanocomposite with a differential scanning calorimeter (Netzsch DSC-200 F3) up to 260 ℃.

#### Thermogravimetry analysis

The thermogravimetry analysis of the powder sample of the nanocomposite has been recorded with a thermal analysis instrument (METTLER TOLEDO, TGASDTA 851e) up to 700 ℃.

#### SEM imaging and EDX spectroscopy

The mesh 100 powder of the nanocomposite has been used for surface imaging with an instrument (TESCAN, MIRA3 FEG-SEM). Also, the instrumentation has been used for elemental mapping and energy dispersive X-ray spectroscopy of the nanocomposite.

#### Dye removing process

Samples have been prepared in vials with 5 ml dye solutions. The effect of different parameters such as the amount of absorbent, pH of the solution, process time, temperature, and dye concentration have been studied. A double beam spectrophotometer (Analytic Jena SPECORD 250) was used to determine the concentration of the removed dye from the solutions. The calibration standards have been obtained from the stock solution with 50 ppm of Congo red. The max wavelength has been recorded at 493 nm for Congo red. The time dependency of the adsorption process has been studied for up to 180 min, and the equilibria adsorption time has been evaluated which was 45 min. Also, the evaluated data is used to calculate the isotherms parameters of adsorption using Langmuir, Freundlich, and Temkin models. Finally, the recovering ability of the nanocomposite has been examined by rinsing 48 h in ethanol and 24 h in distilled water after 5 cycles of the optimized dye removal process, and the efficiency has been evaluated.

## Conclusion

The micelle of the IL 1-octyl-3-methyl imidazolium bromide has been used to prepare a stable aqueous TiO_2_ nanofluid. The stable aqueous micelle nanofluid has been used to prepare the TiO_2_@PVA nanocomposite using the phase inversion process. It should be noted that the ionic liquids in the CMC had a limitation to suspend the TiO_2_ particles related to the water molecules activity, and the annealing process should be carried out as long as possible. The dried powder of nanocomposite has a layered semi-crystalline porous structure that could provide a significant specific interface area that has been characterized by FT-IR, XRD, DSC, TGA, and SEM. Accordingly, it has been used for removing Congo red dye from aqueous solution with significant efficiency that investigations show that the adsorption process could be modeled with Langmuir isotherm theory which means the adsorption is monolayer adsorption on a homogenously distributed surface. Also, the optimization of the process shows that they require time for the best results is 45 min at a higher pH level at a lower temperature using the proportional amount of 0.002 g TiO_2_@PVA/ppm Congo red.

## Data Availability

The COSMO-SAC calculations have been carried out using free benchmark of COSMO-SAC model usnistgov/COSMOSAC repository, [https://github.com/usnistgov/COSMOSAC].
